# Interstate Reciprocity

**Published:** 1903-06

**Authors:** 


### Editorial.

### INTERSTATE RECIPROCITY.

This subject has become of first importance in the evolution of State laws. When these, governing dentistry, were originally passed in the several States, the idea was then that each legislative body should enact a law satisfactory to the dentists within its State boundaries, and no regard was paid to the individuals outside of these limits who might possibly desire to pass over these boundaries and become practitioners therein. While this may have been an oversight upon the part of the framers of the several laws, and selfishness may not be justly ascribed as a motive, it remains as a fact that the effect has been to limit the value of the dental diploma to the State in which it is issued.

Whatever may have been the motive, the effect has been to confine the practitioners of each State within its legal jurisdiction, and to pass beyond, means a State board examination and possibly, as in New York, a decision as to preliminary education. The man of Pennsylvania may have graduated years ago with honor in dentistry, may have practised a quarter of a century with satisfaction to his patients and credit to himself, but should he desire to change his field of usefulness and establish an office in New Jersey or New York, he is at once served with a notice,—No admittance except by compliance with the provisions of the State law. The man whose health has become broken by the severe northern climate may desire to change to more congenial surroundings elsewhere in the United States, but he is again met with the same un-American answer,—No admittance. This has become such a serious statutory evil that even the sticklers for law, and more law, are beginning to feel the pricks and are themselves anxious to find a way out of the legal morass into which they have been plunged by their unwise legislation.

Complaints have been made of the laws of England and Europe excluding dentists from practice in their several countries. These laws, as a rule, are liberal compared to those framed in this country to debar our own people from practice. The present condition is simply a professional war between the States constituting this federation. It is assumed that each State has a right, under the Constitution of the United States, to arrange its own laws to suit its best interests without regard to those of outsiders, foreign or domestic. It has been assumed that this comes under the provision of police duty of the several States. This may be true, but there is another portion of this Constitution that distinctly prohibits one State from legislating against any other,—"Full faith and credit shall be given, in each State, to the public acts, records, and judicial proceedings of every other State."

There has been no decision given by the United States Supreme Court as to the relation this bears to dental laws and their antagonistic attitude to other States. Until a case is carried to this court of last resort there must be a period of legal uncertainty regarding this whole question. A decision is not probable in the near future, as neither side of the controversy seems anxious to meet the issue.

This being true, there is an evident desire in certain quarters to bring about a more liberal condition of things, but no one seems to know where to begin. The men of one State will say to a neighboring State, in substance, When you raise your entrance standard to ours we will consider the proposition of reciprocity, and not before. Another will reply, Our standard is that of the high schools of our State and the diplomas from these we regard as sufficient. Another will contend that the rule adopted by the National Association of Dental Faculties governs the colleges and, indirectly, the State boards, and between these conflicting views the possibility of coming to an amicable decision is very remote.

Recently the "Philadelphia Dental Club," a dining organization, appreciating the importance of this subject, sent out invitations to presidents of State boards and deans of many dental colleges to meet with them round the banqueting board and then and there to discuss this problem. The result was not altogether satisfactory. The declinations were many, and while a goodly number were present and the time was profitably spent, there seemed to be no well-defined plans to meet the difficulty and to hasten the day when an individual duly accredited in one State should be held equally worthy in any other. While the discussion failed to reach a distinct proposition, it did fully represent the chaotic state of the dental professional mind throughout the country, a condition, if not one of apathy, at least bordering on indifference.

As an important profession we cannot afford to let this matter rest where it is at present. Whether legislation in one State antagonizing the acts of another State be declared constitutional by the highest authority in the future, the present attitude of the several States is a serious obstruction to dental practice, and is contrary to the accepted methods in other lines of interstate policy. The State laws governing dentistry, and at present in force, are an effectual barrier against reciprocity. To the writer the analogy between this condition and a tariff on goods established between the States is very strong. This would not for a moment be tolerated, yet here we have something worse,—a tariff on intelligence and skilled training equally at variance with the liberal ideas inculcated in the formation of the Republic.

The question, What can be done? was not answered at the recent symposium alluded to, nor is it possible to give a final answer here. It seems, however, to be capable of solution upon a compromise basis and upon no other. So long as each State insists upon its educational methods being considered primarily to a settlement, nothing can be done. If New York, through the Regents, will insist on so many "counts" as a preliminary basis, there can never be an harmonious settlement with other States, and New York is simply a typical illustration of all the others. It is evident something will have to be sacrificed to promote harmony, for the situation has become intolerable.

While each one may have a solution of the problem, it may be not inappropriate for the writer to give his ideas upon a subject of deep interest not alone to him, but to all dental educators.

In the first place, it would seem to be the duty of the National Association of Dental Examiners to take up this subject seriously at its next meeting at Asheville, N. C. It will hardly be possible, in a large body like this, to consider this important subject as it deserves; the writer would therefore suggest that this body might call a conference of all the presidents of State boards and with these, the deans of Dental Colleges, to consider ways and means to accomplish the end desired. This would seem to meet all the preliminary requirements, and would avoid frictional criticism on the part of colleges or State boards. There is an equal interest upon this subject in colleges and State boards, and both should be consulted.

The writer feels that whatever may be the outcome, there is but one settlement possible, and that is for each State to change its law so that a diploma representing dental college work and State examination should be accepted without question in any State of the Union. To attempt to go back of the State examination and say to the applicants, Your preliminary training has not equalled our requirements, or, The colleges from which you have received your diplomas are not of equal standard with ours, will make interstate reciprocity impossible. The minimum standard of entrance qualifications has been established by the National Association of Dental Faculties, and upon that the diploma has been granted, and upon that basis alone the diploma, supplemented by a State examination, should be universally accepted. Anything more than this cannot reasonably be required. State prejudices must go and be sacrificed to the good of the greatest number, and eventually to the benefit of the dental profession.

